# Self-Regulation in Adolescents: Polish Adaptation and Validation of the Self-Regulation Scale

**DOI:** 10.3390/ijerph19127432

**Published:** 2022-06-17

**Authors:** Maja Gajda, Agnieszka Małkowska-Szkutnik, Wojciech Rodzeń

**Affiliations:** 1 Department of Biomedical Foundations of Development and Sexology, Faculty of Education, University of Warsaw, 00-561 Warsaw, Poland; amalkowska@uw.edu.pl; 2 Institute of Psychology, University of Szczecin, 71-017 Szczecin, Poland; wojciech.rodzen@usz.edu.pl

**Keywords:** self-regulation, measurement, adolescence, adolescent health, factor analysis

## Abstract

Self-regulation is associated with life satisfaction, well-being, and life success. For adolescents, who may be exposed to peer pressure and engage in risky behaviors, the ability to self-regulate or control emotions, thoughts, and behaviors is crucial for healthy development. While self-regulatory skills have long been recognized as important for many areas of life, instruments to measure self-regulation remain limited, especially in Poland. The aim of this study was to adapt and validate the Self-Regulation Scale in the Polish adolescent sample. The data for this study were obtained as part of the Health Behavior in School-aged Children 2021/2022 pilot study. Exploratory and confirmatory factor analyses confirmed that the instrument has satisfying psychometric properties. A three-factor structure of the instrument was obtained with cognitive, behavioral, and emotional subscales, which corresponds to the original instrument and theoretical assumptions. The final version of instrument contains 24 items, and based on the statistical analysis, it is concluded that it is suitable to be used in adolescent samples.

## 1. Introduction

Self-regulation is defined as the ability of individuals to control their behavior, emotion, thoughts, and attention in order to achieve goals [1,2]. Apart from the broad definition, self-regulation is also defined in terms of its specific domains, such as cognition or emotions. This plurality of research approach stems from the fact that there are several different self-regulation models [2,3]. Most models focus on cognitive functioning, motivation, setting goals, and self-monitoring, e.g., Social-Cognitive Theory [4], Goal Systems Theory model [5], and Self-Regulated Learning models [6,7,8]. In contrast, Trait Models [9,10] have in their core emotional regulation skills and abilities. In Trait Models, individual differences in self-regulation are explained by differences in personality traits like impulsivity and conscientiousness. More recently, there have been several attempts to integrate self-regulation models [1,2,11,12]. For example, the Self-Regulation Promotion Model [1] defines self-regulation as a multidimensional construct that subsumes emotional, behavioral, and cognitive dimensions.

Despite differences in the conceptualization of self-regulation, there is a strong consensus that self-regulatory skills play an important role in many areas of life. For instance, a high level of self-regulation is positively related to school achievement [13,14,15]. The ability to self-regulate is also associated with well-being and mental health [16,17,18,19]. In contrast, people with poor self-regulation skills can be more vulnerable to external stimuli like social stressors [20] and more likely to fall into addictions [21,22] and psychosomatic diseases [23].

### 1.1. Research on Self-Regulation Development

Self-regulation is a “normative developmental process” influenced by “biopsychosocial factors” [1]. It means that every individual develops self-regulation with age. The trajectory of this development is determined by temperament but can also be stimulated by social contexts and environmental factors [24,25]. The influence of the environment on self-regulation is especially prominent in early childhood and adolescence. In early childhood, self-regulation develops dynamically through co-regulation and modeling from caregivers. Role models with high self-regulation skills who offer a warm, responsive, and supportive environment are important at that stage [26]. During adolescence, intense social interactions with peers and romantic partners have a further impact on the development of self-regulation skills [24].

Adolescents’ self-regulation tends to be researched less often than self-regulation in young children, however, it is worth studying for several reasons. First, rapid brain maturation in adolescence allows for more complex cognitive processes [27]. Adolescents develop the ability to foresee consequences, plan compound tasks from a long-term perspective, and reflect on their own emotional states [28,29]. As thinking, planning, metacognition, and self-monitoring are very important to self-regulation, the development of these skills is an interesting research problem from the perspective of self-regulation. Second, adolescents are more likely than younger children to engage in binge drinking, illegal substance use, or unprotected sexual behaviors, and high self-regulation is considered a protective factor against those risky behaviors [30]. Studying self-regulation in adolescents can help us understand this mechanism better. Third, adolescents have many important developmental tasks to fulfill, and self-regulation helps them in accomplishing that. High self-regulation skills are important for building lasting friendships and romantic relationships and entering new social roles in adaptive ways [24,31]. To sum up, self-regulation plays an important role in many areas of adolescents’ life, and in adolescence, there are many interesting developmental changes that influence self-regulation, which makes self-regulatory skills in adolescence worth studying.

### 1.2. Background of the Study

For this research, we adopted two main assumptions. First, we focused our study on adolescence, which is a “turning point for self-regulation” [32]. There are many reasons to study self-regulation in adolescents, yet this topic seems insufficiently researched compared to self-regulation in early childhood, for example. Second, we assumed that self-regulation is a multidimensional construct composed of emotional, cognitive, and behavioral dimensions [33,34,35,36]. Studies based on this approach can provide an important insight into an individual’s self-regulation processes [37] but are relatively small in number [38]. To fill this research gap, we adapted and validated a tool that assesses emotional, cognitive, and behavioral self-regulatory skills in adolescents. To our knowledge, this is the first instrument in Polish for measuring self-regulation composed of emotional, behavioral, and cognitive factors.

### 1.3. The Aim of the Study

The Self-Regulation Scale has not been validated in the Polish context so far. Therefore, the aims of our study were twofold (1) to adapt SRS to the Polish context, (2) to establish the factor structure of the Self-Regulation Scale by Novak and Clayton, and to test its psychometric properties, internal consistency, and construct validity. The additional aim was to analyze the distribution of variables and self-regulation scores in the studied population by sex and age.

## 2. Materials and Methods

### 2.1. Review of Self-Regulation Questionnaires

The researchers have developed different approaches to translating self-regulation models into measurable variables [4,5,6,7,8,9,10]. As a result, there is a profusion of tools that assess specific domains of self-regulation. In this section, we present self-regulation tools that have been validated in adolescents.

The majority of tools for adolescents focus on cognitive aspects of self-regulation, such as learning strategies, searching for and working with information, planning, setting goals, self-monitoring, and self-assessment. Examples of such tools are Deep Learning Strategies Questionnaire (DLS-Q), Self-Regulation of Learning Self-Report Scale (SRL-SR), Self-Regulatory Inventory (SRI), and more. Several cognitive-oriented tools also tap emotions related to learning. For example, the Motivational Strategies for Learning Questionnaire (MSLQ) has a subscale measuring test anxiety, and the Emotion and Motivation Self-Regulation Questionnaire (EMSR-Q) measures “emotional and motivational strategies in educational contexts” [39]. Many cognitive-oriented tools, however, overlook the emotional dimension of self-regulation.

Another important factor in self-regulation research is motivation. The role of motivation is pronounced in cognitive-oriented tools used in the educational context. However, there is one interesting motivation-related tool outside this context called the Prosocial Self-Regulation Questionnaire (SRQ-P). The SRQ-P measures the motivation behind behavioral regulation and aims to determine why adolescents refrain from potentially antisocial behaviors and follow socially acceptable ones instead.

To measure emotion regulation, one can choose from a wide range of tools that focus on emotions related to different situations and contexts. The majority of emotion regulation tools refer to emotions related to the self and regulated by the self (intrapersonal emotion regulation), for example, the Cognitive Emotion Regulation Questionnaire (CERQ). There are also tools oriented to emotions evoked by and regulated in social interactions (interpersonal emotion regulation), an example of this kind of tool is the Interpersonal Emotion Regulation Questionnaire (IERQ).

When it comes to measuring the overall aptitude of self-regulation understood as a dynamic between emotional, behavioral, and cognitive dimensions, there are several tools that can be used. Among them are the Dysregulation Inventory [40] intended to assess substance use disorder risk, and the Adolescent Self-Regulatory Inventory [41] focusing on long and short-term self-regulation capacities. In our study, we chose the Self-Regulation Scale [20] for further analysis. We decided to use SRS for several reasons. First, it is composed of three subdimensions that apply to the general or holistic understanding of self-regulation as a feature that is reflected in all aspects of human functioning-thoughts, actions, and emotions. In SRS, emotional and behavioral subscales are reverse scored, and the overall score of the scale indicates the general level of self-regulation. Second, SRS was created for adolescents in the first place, and it is also a good choice because it has a relatively small number of items, which makes it more suitable for young respondents. The brevity of instruments is especially important in long questionnaires such as the HBSC study.

The table below (Table 1) presents a list of self-regulation self-report questionnaires grouped by domains (cognitive, emotional, etc.). The table consists of information about authors, country, year of publication, number of items in the tool, and subscales.

### 2.2. Methods

The study had a quantitative design and was conducted in June 2021 among Polish adolescents who attended primary and secondary schools. The participants filled in paper-pencil questionnaires as part of the Health Behavior in School-Aged Children (HBSC) pilot study. The HBSC (Health Behavior in School-aged Children) research network is an international alliance of researchers that collaborate on the cross-national survey of school students. The study is conducted every four years. Poland has been part of the international network since 1989. The data for this study were obtained as part of the HBSC 2021/2022 pilot study). The study was coordinated by two institutions: the Faculty of Education of the University of Warsaw, and the Institute of Mother and Child (Poland). The approval of a Bioethics Committee was obtained for the study (number: 51/2021).

The Polish version of the 26-items SRS was created with the back-translation technique recommended for cross-cultural validation [42]. Original SRS was translated to Polish by a professional translator, then it was back-translated into English by another professional. Finally, the original and the back-translated versions were compared and revised by independent specialists to ensure coherence and semantic consistency. The translated questionnaire was consulted with a group of 10 adolescents to ensure that the wording used in the statements was comprehensible and easily interpreted by the targeted age group.

### 2.3. Participants

The sample consisted of 392 adolescents aged 11 to 17 (M = 14.33; SD = 1.77). There were 169 boys (43.1%) and 223 girls (56.9%). 170 participants (43.4%) indicated urban areas as their place of residence, and 222 participants (56.6%) responded that they lived in rural areas. When it comes to education there were 228 participants (58.1%) who attended primary school, and 164 participants (41.9%) attended secondary school. Additionally, participants were divided into two subgroups based on the developmental stages of adolescence: early adolescence with 262 (66.8%) respondents from 11 to 13-years old (M = 12.29; SD = 0.79), and middle adolescence with 130 (33.2%) respondents from 14 to 17-years old (M = 15.34; SD = 1.13).

### 2.4. Measures

#### 2.4.1. Self-Regulation Scale (SRS)

The Self-Regulation Scale (SRS) used in the study is a self-report tool that has 26 items falling into three subscales. It was created with adolescents in mind and based on a similar tool used by Dawes and Kendall [43,44]. SRS contains statements about controlling emotions (*I have difficulty controlling my temper*), and behavior (*I get very fidgety after a few minutes if I am supposed to sit still*), setting and achieving goals (*I develop a plan for all my important goals; I put my plans into action*), and staying focused on tasks (*I have difficulty keeping attention on tasks*). Respondents rate how true each statement is for them on a 4-point scale ranging from 1 (Never true) to 4 (Always true). The statements for the emotional and behavioral dimensions are scored inversely. Higher scores show better self-regulation skills. In their original research, Novak and Clayton found internal consistency scores of α = 95 for emotional, α = 96 for cognitive, and α = 94 for behavioral dimensions.

#### 2.4.2. Measures Used for Validation

The SRS was correlated with four variables selected from the HBSC Pilot Study. The following measures from HBSC Questionnaire were used to assess the construct validity of SRS:

##### Cantril Ladder

Cantril Ladder [45] is a self-report instrument for measuring overall satisfaction with life. It is a 1-item scale with 10 points, where 0 means the worst possible life and 10 means the best possible life. The respondents are asked to mark the ladder step that describes best where they feel they stand. In the HBSC study, there were some minor wording changes introduced to make the description easy to understand for adolescents [46].

##### WHO-5 Well-Being Index

The WHO-5 is a 5-item instrument used for measuring well-being. It taps positive mental states, such as relaxation, good mood, and positive energy experienced by respondents in the past two weeks. The respondents are asked to indicate for each of five statements how often they have felt a given emotion over the last two weeks on a 6-point scale (1. All the time, 6. At no time.). The WHO-5 is suitable for children 9 and above. Additionally, it is a screening tool for depressive symptoms [47]. In this study, Cronbach’s alpha reached 0.85.

##### Generalized Anxiety Disorder

Generalized Anxiety Disorder questionnaire [48] was used. It is a brief measure for assessing feelings of anxiety experienced in the last 14 days. The GAD-7 is a 4-point scale (1—Not at all, 4—Nearly every day) that has been validated in adolescent samples [49] and demonstrated good psychometric properties. In our study, Cronbach’s alpha reached 0.90.

##### General Self-Efficacy

The short version of the General Self-Efficacy [50] was used to assess respondents’ self-beliefs on their ability to plan, organize, and follow through with actions in the face of difficulties, challenges, and stressful situations. The original scale was developed by Matthias Jerusalem and Ralf Schwarzer in 1981. The short version used in HBSC study consists of two statements (How often do you find a solution to a problem if you try hard enough? How often do you manage to do the things that you decide to do?) with answers on a 5-item Likert scale (1-Never, 5-Always). In this study, Cronbach’s alpha reached 0.70.

## 3. Results

### 3.1. Statistical Analysis

All analyses were performed using IBM SPSS Statistics 26.0 and IBM SPSS AMOS 21.0 with a 95% confidence interval. Prior to exploratory and confirmatory factor analysis (EFA, CFA), data were verified in terms of removing all missing data, and a factor analysis assumption test was performed.

#### 3.1.1. Preliminary Analysis

To test the EFA assumptions, analyses were carried out using statistics showing the measures of the obtained correlation matrix (the Kaiser–Meyer–Olkin (KMO) test) for sampling adequacy and the Bartlett’s sphericity test.

Based on the results obtained from the descriptive statistics of all variables included in the analyzed structure of the tool (Table 2), it was determined that their distribution significantly differed from the normal distribution (*p* < 0.001), however, the values of skewness and kurtosis themselves ranged from −2 to +2 [51], therefore the deviation was not significant. As a result, it was decided to calculate factor loadings using the principal axis method, which does not require the analyzed variables to have a normal distribution [52]. In addition, the extraction communalities determining the variance of the given variables obtained values greater than 0.3, which indicated the possibility of assigning all variables to the structure of the tool [53].

The KMO test that compared the zero-order correlations to the partial correlations between pairs of variables (Munro, 2005) was equal to 0.88. As a result, the Kaiser (1974) composition was satisfied that the value of this test should be greater than 0.5. The statistically significant value of the Bartlett’s sphericity test (χ^2^(325) = 4014.67; *p* < 0.001) indicates significant correlation coefficients included in the matrix and the presence of enough shared variance [54]. The anti-image correlation matrix contained the correlation values of individual items with a total score ranging from 0.83 to 0.93 which exceeded the border value of 0.5, indicating the need to remove a given variable from further analyses [55].

#### 3.1.2. Exploratory Factor Analysis

To determine the internal structure of the tool, an exploratory factor analysis (EFA) was performed using the principal axis method with extraction method based on eigenvalue value greater than 1 and oblique rotation direct Oblimin with Kaiser normalization with the critical factor load value below 0.4 [53], due to theoretical basis of the original tool proving the possibility of the correlation of the factors [52].

**Figure 1:**
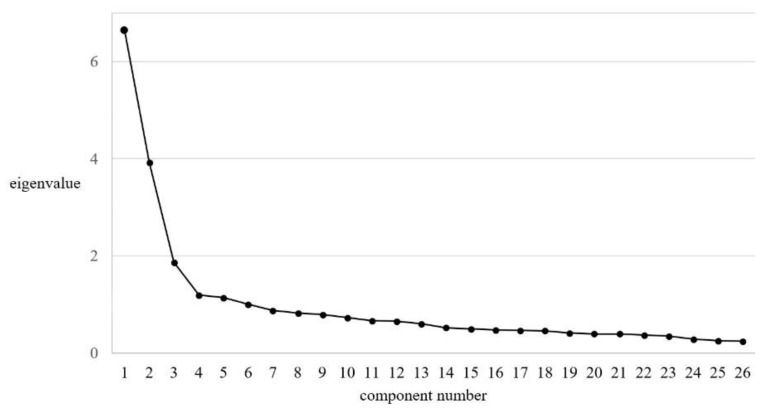
Scree plot of eigenvalues.

The first step to estimate the number of extracted factors was the analysis of the scree plot (Figure 1) and the value of total extraction sums of squared loadings and the percentage of explained variance by the extracted factors.

Based on the analysis of the scree plot, it can be concluded that three factors assumed eigenvalues greater than other factors, however, in terms of distinguishing factors using the principal component axis method, six factors were observed, which explained about 50% of the variance. However, only three factors assumed the sum of the squared loadings greater than 1, which is considered a critical value [56]. As a result, it was decided to re-run the EFA, extracting the forced number of three factors in line with the original design of the tool. The results (Table 3) indicated that about 49% of the variance was explained by the factors obtained, which had an eigenvalue greater than 1, which is an acceptable criterion [55,56].

After oblique direct Oblimin rotation, it was shown that item SR_25 (I get bored/restless easily) was loading the first (0.43) and third (−0.39) factors simultaneously, while item SR_19 (I spend money without thinking about it first) had a factor load value of less than 0.4 (0.36). As a result, it was decided to remove these two statements from further analyses.

The final model matrix (Table 4) presented the identification of three factors, the statements of which had factor loadings higher than 0.4 and were assigned in terms of content to dimensions identical to the original tool. As a result, factor loadings of 9 statements specific to the first factor (emotional dimension) ranged from 0.44 to 0.77, another 9 statements assigned to the second factor (cognitive dimension) had loadings that ranged from 0.58 to 0.75, and the structure of the last factor (behavioral dimension) contained 6 items with the values of factor loadings from 0.52 to 0.84.

The items were assigned to the three subdimensions—emotional, behavioral, and cognitive—in a way that corresponds with the assumptions of the original tool. Emotional dimension describes a propensity to experience intense emotions such as anger (*I fly off the handle for no good reason*) and frustration (*I get so frustrated that I often feel like a bomb ready to explode*), and falling into interpersonal conflicts (*I get into arguments when people disagree with me*). Behavioral dimensions represent fidgetiness (*I get very fidgety after a few minutes if I am supposed to sit still*) and being in movement (*I have difficulty remaining seated at school or at home during dinner),* as well as difficulties in focusing and directing attention (*Most of the time, I don’t pay attention to what I am doing; I have difficulty keeping attention on tasks*). Cognitive dimension indicates planning skills (*I develop a plan for all my important goals*), foreseeing consequences (*I think about the future consequences of my actions*), reflectiveness (*I think about my mistakes to make sure they don’t happen again*), and monitoring situation (*As soon as I see things are not working out, I do something about it*).

In the next step of the analysis, statistically significant values of Pearson’s r correlation coefficients between the selected factors were obtained (Table 5). The emotional dimension correlated positively and statistically significantly with the behavioral dimension (r = 0.41; *p* < 0.01) and negatively with the cognitive dimension (r = −0.11; *p* < 0.05). The correlation coefficient between the cognitive and behavioral dimensions was r = −0.18 (*p* < 0.05).

#### 3.1.3. Confirmatory Factor Analysis

**Figure 2:**
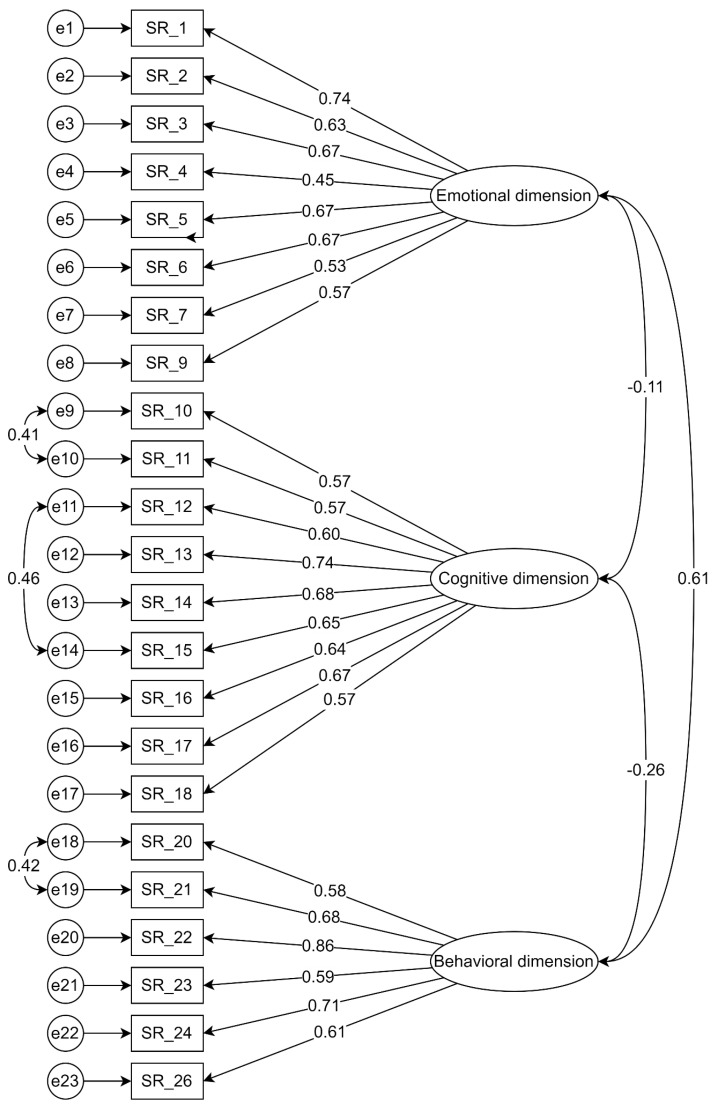
Confirmatory factor analysis (CFA) model for the questionnaire assessing self-regulation.

Based on the internal structure of the tool obtained after the EFA, the same factor structure was checked using the structural model confirmatory factor analysis (CFA) with the maximum likelihood estimation method. On the basis of the adopted criterion excluding the statements assuming standardized factor loadings values less than 0.4, the statement SR_8 (“I slam doors when I am mad.”), assuming a factor load equal to 0.32, was removed from the structure of the tool. The final values of the factor loadings of the accepted theorems ranged from 0.45 to 0.86. In addition, based on the analysis of the covariance matrix between item errors, three covariance paths between pairs of errors were added in the same dimensions to constrain the redundancy effect and increase the model fitness: e9–e10, e11–e14, and e18–e19, which allowed for further analysis [57] of the obtained model (Figure 2) in terms of fit indices (Table 6).

For the analyzed model, a statistically significant value of the chi-square test (χ^2^(222) = 469.60; *p* < 0.001) was obtained, however, it is a frequent phenomenon with a large sample size [58,59]. Moreover, the relative/normed chi-square value (χ^2^/df = 2.12) took an acceptable value below 5.0 [60].

Most of the other fit indices turned out to be acceptable (Table 6). Thus, for the final model of the tool, the value of the goodness-of-fit index (GFI = 0.90) and the comparative fit index (CFI = 0.93) were obtained, exceeding the permissible value of 0.9 [61,62], but a different result was obtained for adjusted goodness-of-fit (AGFI = 0.88) and normed-fit index (NFI = 0.87), which minimally did not meet the expected criterion 54,61. Root mean square residual index (RMR = 0.05) is also acceptable [63]. However, the last of the selected coefficients-root mean square error of approximation index (RMSEA = 0.05) was below 0.08, which proves a good fit [64]. Moreover, its value turned out to be statistically insignificant (*p* = 0.20; 90CI [0.05; 0.06]).

On the basis of the obtained values of the fit indicators, it can be concluded that this model was well suited to the data, mostly at an acceptable level. The obtained values could be mainly influenced by the large sample size [65]. Additionally, the questionnaire is characterized by a high Cronbach’s alpha reliability coefficient for the overall score (α = 0.86) and three dimensions of self-regulation-emotional (α = 0.83), cognitive (α = 0.86), and behavioral (α = 0.84).

#### 3.1.4. Convergent Validity

To determine convergent validity, analyses were performed using Pearson’s r correlation coefficient to estimate the level of correlation between the three dimensions of the adapted tool and the overall result of self-regulation and four selected variables from the HBSC studies—well-being, life satisfaction, anxiety, and general self-efficacy (Table 7).

The obtained results indicate a statistically significant correlation between the analyzed variables. The overall level of self-regulation correlated significantly and positively with general self-efficacy (r = 0.52; *p* < 0.01), well-being (r = 0.46; *p* < 0.01), life satisfaction (r = 0.34; *p* < 0.01), and negatively with anxiety (r = −0.45; *p* < 0.01). Similar results were obtained for the correlation of selected variables with individual dimensions of self-regulation. Only the cognitive dimension did not correlate, in a statistically significant way, with anxiety (r = −0.05; *p* > 0.05). Moreover, all three dimensions correlated strongly and positively with the overall level of self-regulation: emotional (r = 0.71; *p* < 0.01), cognitive (r = 0.68; *p* < 0.01), behavioral (r = 0.75; *p* < 0.01), which proves that higher results obtained for individual factors indicate a higher level of self-regulation.

To confirm obtained results and enhance the internal validity of the study by limiting the influence of confounding and extraneous variables, a hierarchical regression analysis with control variables was performed (Table 8).

In the first step of the hierarchical regression analysis, control variables were entered (age, gender, area of residence). In the second step, each of the outcome variables were added as an individual predictor of general self-regulation. After considering the effects of the control variables, self-regulation turned out to be significantly related to four measured variables: general self-efficacy (β = 0.51; *p* < 0.001), well-being (β = 0.49; *p* < 0.001), life-satisfaction (β = 0.36; *p* < 0.001), and anxiety (β = −0.47; *p* < 0.001). Additionally, the obtained values of the standardized regression coefficients are similar to the obtained correlation coefficients.

### 3.2. Self-Regulation among Polish Adolescents

An additional aim of the study was to describe the general level of self-regulation and its three dimensions in the entire research sample (Table 9), and among the subgroups in terms of the measured demographic variables (Table 10). Unfortunately, due to significantly unequal subgroups regarding gender (χ^2^ = 7.44; *p* < 0.01), stage of development (χ^2^ = 44.45; *p* < 0.001), place of residence (χ^2^ = 8.58; *p* < 0.01), the comparisons were limited to the analysis of basic statistics descriptive of the level of self-regulation among particular groups. Regarding the stage of development, self-regulation was analyzed in two subgroups based on adolescence development—early adolescence (10–13 years old) and middle adolescence (14–17 years old).

In the total sample, the highest mean value in terms of tool factors was obtained for the cognitive dimension (M = 24.83; SD = 5.64), lower for the emotional dimension (M = 23.71; SD = 4.71), and the lowest for behavioral dimension (M = 17.34; SD = 4.09). The mean general level of self-regulation was 65.88 (M = 65.88; SD = 10.28).

In the study, a higher mean of self-regulation results was observed among boys (M = 64.68; SD = 9.28), middle adolescents (M = 66.67 SD = 10.22), and those from urban areas (M = 66.07; SD = 10.50) compared to girls (M = 64.51; SD = 10.79), early adolescents (M = 64.27; SD = 10.22), and adolescents from rural areas (M = 65.73; SD = 10.13). Moreover, boys achieved higher mean scores than girls in the emotional (M = 25.40; SD = 4.10) and behavioral (M = 17.57; SD = 3.99) dimensions, and the girls had a higher cognitive factor score (M = 24.92; SD = 5.44) than boys (M = 24.71; SD = 5.91). Early adolescents showed a higher mean of emotional dimension score (M = 24.05; SD = 4.68) than middle adolescents (M = 23.53; SD = 4.73), while middle adolescents achieved a higher level of cognitive dimension (M = 25.70; SD = 5.31) compared to early adolescents (M = 23.07; SD = 5.89). Additionally, middle adolescents showed a higher average behavioral level (M = 17.44; SD = 3,97) than early adolescence (M = 17.15; SD = 4,33). Students from urban areas were characterized by a higher average level of the cognitive (M = 25.56; SD = 5.30) and behavioral (M = 17.41; SD = 4.01) dimensions and a lower level of the emotional dimension (M = 24.16; SD = 4.57) than students from rural areas.

The age correlated positively and weakly, but was statistically significant with the cognitive (r = 0.26; *p* < 0.01) and emotional (r = 0.10; *p* < 0.05) dimensions, and the general level of self-regulation (r = 0.10; *p* < 0.05). There was no correlation between age and the behavioral dimension of self-regulation (r = 0.03; *p* > 0.05).

## 4. Discussion

The main aim of the study was to examine the psychometric properties of the Polish Self-Regulation Scale. Based on the results of the exploratory factor analysis, it was necessary to eliminate two statements due to the unsatisfactory factor load values. Despite this, the created three-factor structure of the tool was both consistent with the assumptions of the original model and explained about 49% of the total variance. This value did not exceed the satisfactory value of 50–60% of the explained variance [55,66,67] but the first factor explained more than 25% of the variance, while the last one had a value greater than 5% [55,68], which is considered an acceptable value and indicates a practical and scientifically useful use of the questionnaire. Moreover, Preacher and MacCallum postulate that large loadings may denote even around 30–40% [69]. However, factors loaded in line with the conceptualization of the original tool’s hypothesized research model, and Cronbach’s alpha coefficient reached 0.86, which indicates that the Polish version of the tool can be successfully subjected to further validation process. Additionally, it is worth noting that in a Chinese validation study of the same tool, EFA “yielded a three-factor solution accounting for 50.39% of the total eigenvalues” [70], which is a result very similar to ours. Close proximity of the two outcomes may indicate that perhaps the low variance results from the specificity of the tool, or it may be connected with the samples and the quality of data collected in those two studies.

The results of the confirmatory factor analysis after removing one of the items and matching the three covariance paths between errors confirm the structure of the tool obtained on the basis of EFA and indicate satisfactory values of fit indices. The need to introduce covariance paths may have been due to the presence of certain items in the model which were redundant to each other. Doing covariation between the measurement errors of redundant items constrained the redundancy effects and thus improved the overall model fit. The tool was characterized by the preferred [71] and reliable [72] values of the Cronbach’s alpha coefficient ranging from 0.84 to 0.86 for individual factors and 0.86 for the overall score. Taking into consideration the results of the conducted factor and reliability analyses, it can be concluded that the Polish version of the self-regulation questionnaire presents satisfactory psychometric properties, which indicates its practical application and further usefulness in research on self-regulation.

In the next step of the study, we validated the P-SRS with four instruments measuring adolescents’ mental health—Cantril Ladder, Who-5 Well-Being Index, Generalized Anxiety Disorder, and General Self-Efficacy. The results of validation showed that the overall level of SRS correlated statistically significantly and positively with general self-efficacy, life satisfaction, and well-being, yet negatively with anxiety. All those findings are consistent with previous studies. For example, Bouffard-Bouchard et al. observed that students with high self-efficacy were better at self-regulating while performing tasks. The participants showed greater task persistence and better time management skills [73]. Calmeiro and colleagues found that self-regulation was a significant predictor of life satisfaction [74]. Additionally, Singh and colleagues found that self-regulation was significantly and positively correlated with overall well-being and in particular with its dimensions, i.e., personal growth, personal relatedness, purpose in life, and self-acceptance [75]. Kocovski and Endler observed a significant negative correlation between self-regulation and social anxiety [76]. High social anxiety is related to negative emotions about the self, and it also correlates with low self-regulation skills. As shown in the previous studies, self-regulation is important for well-being, life satisfaction, and self-efficacy.

An additional aim of our study was to analyze self-regulation levels in the entire research sample and in terms of demographic variables. One interesting finding from the analysis is that younger participants aged 11–13 scored higher on the emotional dimension of self-regulation than their older peers in the middle adolescence group. In general, it is acknowledged that self-regulation develops with age and children become more skillful at self-regulation as they transition into adolescence and adulthood [77]. For example, Sanchis-Sanchis et al. found that children aged 13–16 years obtained higher scores in the emotional regulation strategies than the 9–12 year group [78]. The difference in the obtained results may be explained by the tools and items used to measure emotion regulation. Older adolescents are likely to know more strategies to stay calm in emotionally challenging moments than their younger peers, as noted in Sanchis-Sanchis’ study. However, at the same time, experiencing anger becomes more complex in adolescence [79]. Additionally, as reported by Deno et al., adolescents aged 15–16 express their frustration and anger more openly and violently than 10 years old children [80]. Violent anger expression intensifies in middle adolescence [81], which may be the reason for the lower self-regulation scores of middle adolescents in our study.

## 5. Conclusions

The following article presents the results of the adaptation and validation of the Self-Regulation Scale in an adolescent sample. Our study was, to our knowledge, the first attempt to adapt and validate an instrument for measuring self-regulation in the Polish youth population. On the basis of the obtained results of EFA and CFA, it can be concluded that the final structure of the tool is not perfectly proportional to the original structure, however, the obtained psychometric properties are satisfying, and the tool itself can be successfully used in scientific practice in the Polish context and be subjected to further validation processes. Moreover, the obtained three-factor structure with cognitive, behavioral, and emotional factors is an optimal solution corresponding to the theoretical and empirical assumptions of the adapted tool.

Promoting self-regulation may be helpful in preventing risk behaviors and mental health problems in adolescents [30,82,83], as well as facilitating prosocial behaviors [84]. Studying adolescents’ self-regulation can give educators, teachers, and parents the knowledge necessary to actively support the development of self-regulatory skills in adolescence. Further studies on self-regulation are needed, especially in countries where self-regulation is not a leading research problem. To conduct methodologically sound research, adapted and validated tools are necessary. The results of the current study can be used by other researchers to study self-regulation in early and middle adolescence in the Polish context, as the instrument was validated in students aged 11–17.

## 6. Limitations

The study has several limitations. For example, dependent variables used for the validation of the instruments were chosen from a predefined set of variables included in the HBSC pilot study. Further studies should include a wider selection of variables. Moreover, the HBSC study employs cross-sectional data and not time-series cross-sectional data. The adopted methodology limited the potential analyses when it comes to a behavioral change over a period of time. Additionally, the cross-sectional design makes it impossible to draw cause-and-effect conclusions. Future stages of the validation of this instrument should be based on longitudinal studies to fully determine the validity of the questionnaire. A longitudinal design of the study would provide an opportunity to measure self-regulation as a trait and track its changes at the different life stages of the participant from a representative sample. This approach would allow the instrument to be standardized and used further in diagnostic and clinical practice.

One of the limitations of the study was a research sample size [85] that was not properly differentiated in terms of age, which made it impossible to determine whether the differences between the age groups were statistically significant, e.g., using the T-test or analysis of variance. Because of that, we decided to limit the presented data to the mean and standard deviation for the early and middle adolescence subgroups. Additionally, in our study, we did not include the late adolescence group, which could provide valuable comparisons. We think that future research should ensure that the tool is validated in the Polish population, taking into account a more diverse and representative sample, particularly in other age groups. Moreover, another limitation is disproportions in the subgroups in terms of measured demographic variables, such as gender, which made it impossible to analyze the significance of differences in the average level of self-regulation among boys and girls. Additionally, in this study, we investigated the 26-item instrument, and in further analyses, a short version of the scale may be considered due to the usefulness of shorter scales, especially with non-adult samples.

## Figures and Tables

**Table 1:** Self-regulation self-report measures used in adolescent samples.

Tools	Authors (Country)	Year	Items	Subscales
Cognitive self-regulation or/and emotion regulation in educational contexts				
Deep Learning Strategies Questionnaire (DLS-Q)	Panadero, Alonso-Tapia, García-Pérez, Fraile, Manuel Galán, and Pardo (Spain)	2021	30	(1) Basic learning self-regulation strategies (2) Visual elaboration and summarizing strategies (3) Deep information processing strategies (4) Social learning self-regulation strategies
Emotion and Motivation Self-Regulation Questionnaire (EMSR-Q)	Panadero and Alonso-Tapia (Spain)	2020	20	(1) Avoidance oriented self-regulation (2) Negative self-regulation of stress (3) Performance oriented self-regulation (4) Process oriented self-regulation (5) Positive self-regulation of motivation
Self-Regulation of Learning Self-Report Scale (SRL-SR)	Toering, Elferink-Gemser, Jonker, van Heuvelen, and Visscher (Netherlands)	2012	46	(1) Planning (2) Self-monitoring (3) Evaluation (4) Reflection (5) Effort (6) Self-efficacy
Self-Regulation Strategy Inventory–Self-Report (SRSI-SR)	Cleary (United States)	2006	45	(1) Seeking and learning information (2) Maladaptive regulatory behaviors (3) Managing environment/behavior
The Short Self-Regulation Questionnaire (SSRQ)	Carey, Neal, and Collins (United States)	2004	31	(1) Goal attainment (2) Mindfulness (3) Adjustment (4) Proactiveness (5) Goal setting
Self-Regulatory Inventory (SRI)	Hong and O’Neil (United States)	2001	34	(1) Planning (2) Self-checking (3) Effort (4) Self-efficacy
Self-Regulation Questionnaire (SRQ)	Brown, Miller, and Lawendowski (United States)	1999	63	(1) Receiving relevant information (2) Evaluating the information and comparing it to norms (3) Triggering change (4) Searching for options (5) Formulating a plan (6) Implementing the plan (7) Assessing the plan’s effectiveness
Self-Regulation Scale (SRS)	Schwarzer, Diehl, and Schmitt (United States and Germany)	1999	10	(1) Attention regulation (2) Emotion regulation
Motivational Strategies for Learning Questionnaire (MSLQ)	Pintrich and De Groot (United States)	1990	44	(1) Self-efficacy (2) Intrinsic value, (3) Cognitive strategy use (4) Self-regulation (5) Test anxiety
Academic Self-Regulation Questionnaire (SRQ-A)	Ryan and Connell (United States)	1989	32	(1) External regulation, (2) Introjected regulation, (3) Identified regulation (4) Intrinsic motivation
Emotion regulation				
Interpersonal Emotion Regulation Questionnaire (IERQ)	Hofmann, Carpenter, and Curtiss (United States and Germany)	2016	20	(1) Enhancing positive affect (2) Perspective taking (3) Soothing (4) Social modeling
Adolescents’ Emotion Regulation Strategies Questionnaire	Kostiuk (Canada)	2013	80	(1) Positive emotion regulation strategies (2) Negative body experiences or strategies (3) Social connection (4) Negative cognition strategies
Difficulties in Emotion Regulation Scale-SF (DERS-SF)	Kaufman, Xia, Fosco, Yaptangco, Skidmore, and Crowell (United States)	2015	18	(1) Nonacceptance of emotional responses (2) Difficulty engaging in goal-directed behavior (3) Impulse control difficulties (4) Lack of emotional awareness (5) Limited access to emotion regulation strategies (6) Lack of emotional clarity
Emotion Regulation Questionnaire for Children and Adolescents (ERQ-CA)based on the ERQ (Gross and John, 2003)	Gullone and Taffe (Australia)	2012	10	(1) Reappraisal (2) Suppression
Cognitive Emotion Regulation Questionnaire (CERQ)	Garnefski, Kraaij, and Spinhoven (Netherlands)	2001	36	(1) Self-blame (2) Other-blame (3) Rumination (4) Catastrophizing (5) Positive refocusing (6) Planning (7) Positive reappraisal (8) Putting into perspective (9) Acceptance
Prosocial self-regulation				
Prosocial Self-Regulation Questionnaire (SRQ-P)	Ryan and Connell (United States)	1989	25	(1) External regulation (2) Introjected regulation (3) Identified regulation
General self-regulation				
Adolescent Self-Regulatory Inventory (ASRI)	Moilanen (United States)	2007	36	(1) Short term self-regulation (2) Long term self-regulation
Self-Regulation Scale (SRS)	Novak and Clayton (United States)	2001	26	(1) Emotional (2) Cognitive (3) Behavioral
Dysregulation Inventory (DI)	Mezzich, Tarter, Giancola, and Kirisci (United States)	2001	92	(1) Affect (2) Behavior (3) Cognitive

**Table 2:** Descriptive statistics of variables, the results of the Shapiro–Wilk normality test, and the extraction communalities.

Item	M	SD	Skewness	Kurtosis	W	Ex. Comm.
SR_1	2.42	0.80	0.20	−0.38	0.86 ***	0.70
SR_2	1.88	0.89	0.83	−0.03	0.81 ***	0.59
SR_3	1.89	0.89	0.73	−0.28	0.82 ***	0.58
SR_4	2.06	0.85	0.52	−0.28	0.85 ***	0.32
SR_5	1.63	0.83	1.13	0.41	0.74 ***	0.51
SR_6	2.15	0.81	0.16	−0.68	0.86 ***	0.62
SR_7	2.23	0.97	0.26	−0.95	0.87 ***	0.55
SR_8	1.76	0.88	1.07	0.45	0.77 ***	0.56
SR_9	2.04	0.97	0.50	−0.83	0.84 ***	0.50
SR_10	2.70	1.00	−0.36	−0.91	0.86 ***	0.71
SR_11	2.66	0.87	−0.30	−0.55	0.87 ***	0.70
SR_12	2.85	0.95	−0.44	−0.74	0.86 ***	0.66
SR_13	2.87	0.91	−0.47	−0.54	0.86 ***	0.65
SR_14	2.79	0.83	−0.33	−0.38	0.86 ***	0.62
SR_15	2.73	0.97	−0.32	−0.84	0.87 ***	0.69
SR_16	2.84	0.90	−0.38	−0.65	0.86 ***	0.71
SR_17	2.67	0.91	−0.16	−0.78	0.88 ***	0.59
SR_18	2.71	0.86	−0.18	−0.62	0.87 ***	0.50
SR_19	2.13	0.99	0.54	−0.73	0.85 ***	0.59
SR_20	1.97	0.94	0.71	−0.39	0.83 ***	0.68
SR_21	2.03	0.95	0.55	−0.67	0.84***	0.74
SR_22	2.22	0.87	0.28	−0.59	0.87 ***	0.74
SR_23	1.84	0.91	0.83	−0.23	0.80 ***	0.67
SR_24	2.01	0.82	0.47	−0.32	0.84 ***	0.60
SR_25	2.32	0.90	0.06	−0.84	0.87 ***	0.53
SR_26	2.59	0.96	−0.13	−0.93	0.88 ***	0.45

Note. W—Shapiro–Wilk normality test. *** *p* < 0.001.

**Table 3:** Total variance explained values.

Component	Extraction Sums of Squared Loadings		
	Total	% of Variance	Cumulative %
1	6.01	25.06	25.06
2	3.88	16.18	41.23
3	1.86	7.75	48.98

**Table 4:** Model matrix obtained on the basis of oblique direct Oblimin rotation.

Items	Factors		
	1	2	3
6. There are days when I’m on edge all the time.	0.77		
1. I have difficulty controlling my temper.	0.75		
5. I fly off the handle for no good reason.	0.69		
3. I get so frustrated that I often feel like a bomb ready to explode.	0.69		
2. When I am angry, I lose control over my actions.	0.66		
9. My mood goes up and down without a reason.	0.60		
7. I easily become emotionally upset when I am tired.	0.60		
4. I get into arguments when people disagree with me.	0.50		
8. I slam doors when I am mad.	0.44		
13. Once I have a goal, I make a plan how to reach it.		0.75	
15. I consider what will happen before I make a plan.		0.74	
16. I think about my mistakes to make sure they don’t happen again.		0.73	
17. I spend time thinking about how to reach my goals.		0.72	
12. I think about the future consequences of my actions.		0.70	
14. As soon as I see things are not working out, I do something about it.		0.69	
11. I put my plans into action.		0.63	
10. I develop a plan for all my important goals.		0.62	
18. I stick to a task until it is finished.		0.58	
21. I get very fidgety after a few minutes if I am supposed to sit still.			0.84
23. I can’t seem to stop moving.			0.81
20. I have difficulty remaining seated at school or at home during dinner.			0.77
22. I have difficulty keeping attention on tasks.			0.70
24. Most of the time, I don’t pay attention to what I am doing.			0.58
26. Little things throw me off when I am working/studying.			0.52

Note. 1—Emotional dimension; 2—Cognitive dimension; 3—Behavioral dimension.

**Table 5:** Component matrix.

	EMO	COG	BEH
EMO	1	-	-
COG	−0.11 *	1	-
BEH	0.41 **	−0.18 *	1

Note. EMO—Emotional dimension; COG—Cognitive dimension; BEH—Behavioral dimension. ** *p* < 0.01; * *p* < 0.05.

**Table 6:** Model fit indices.

χ^2^/df	GFI	AGFI	RMR	NFI	CFI	RMSEA
2.12	0.90	0.88	0.05	0.87	0.93	0.05

Note. GFI—goodness-of-fit; AGFI—adjusted goodness-of-fit; RMR—rppt, mean square residual; SRMR—standardized root mean square residual; NFI—normed-fit index; CFI—comparative fit index; RMSEA—root mean square error of approximation.

**Table 7:** The values of the Pearson r correlation coefficients between the individual dimensions of the adapted tool as well as the overall result and selected variables.

	EMO	COG	BEH	SR	LS	WELL	ANX	GSE
EMO	1	-	-	-	-	-	-	-
COG	0.10 *	1	-	-	-	-	-	-
BEH	0.50 **	0.22 **	1	-	-	-	-	-
SR	0.71 **	0.68 **	0.75 **	1	-	-	-	-
LS	0.33 **	0.18 *	0.23 **	0.34 **	1	-	-	-
WELL	0.50 **	0.21 **	0.31 **	0.46 **	0.59 **	1	-	-
ANX	−0.59 **	−0.05	−0.40 **	−0.45 **	−0.41 **	−0.47 **	1	-
GSE	0.24 **	0.53 **	0.29 **	0.52 **	0.34 **	0.41 **	−0.25 **	1

Note. EMO—Emotional dimension; COG—Cognitive dimension; BEH—Behavioral dimension; SR—Self-regulation; WELL—Well-being; LS—Life satisfaction; ANX—Anxiety; GSE—General self-efficacy. ** *p* < 0.01; * *p* < 0.05.

**Table 8:** The values of the hierarchical regression analysis.

Variable	SE B	β	F	R^2^	ΔR^2^
*Step 1*					
Age	0.29	0.10	4.44 **	0.03	
Gender	1.04	0.15			
Place of residence	0.35	−0.01			
*Step 2*					
LS	0.24	0.36 ***	17.45 ***	0.15	0.12
WELL	0.08	0.49 ***	32.85 ***	0.25	0.22
ANX	0.09	−0.47 ***	29.76 ***	0.24	0.21
GSE	0.28	0.51 ***	38.52 ***	0.29	0.26

Note. LS—Life satisfaction; WELL—Well-being; ANX—Anxiety; GSE—General self-efficacy. ** *p* < 0.01; *** *p* < 0.001.

**Table 9:** Mean and standard deviation of the general level of self-regulation and the three dimensions for the total research sample (N = 392).

	Total Sample	
	Mean	SD
EMO	23.71	4.71
COG	24.83	5.64
BEH	17.34	4.09
SR	65.88	10.28

Note. EMO—Emotional dimension; COG—Cognitive dimension; BEH—Behavioral dimension; SR—Self-regulation.

**Table 10:** Mean and standard deviation of the general level of self-regulation and the three dimensions for the subgroups divided by gender (boys vs. girls), developmental stage (early adolescence vs. middle adolescence), and place of residence (rural vs. urban areas).

	Gender			
	Mean		SD	
	Boys	Girls	Boys	Girls
EMO	25.40	22.43	4.10	4.75
COG	24.71	24.92	5.91	5.44
BEH	17.57	17.16	3.99	4.16
SR	67.68	64.51	9.28	10.79
	**Adolescence Stage**			
	Mean		SD	
	Early	Middle	Early	Middle
EMO	24.05	23.53	4.68	4.73
COG	23.07	25.70	5.89	5.31
BEH	17.15	17.44	4.33	3.97
SR	64.27	66.67	10.22	10.23
	**Place of Residence**			
	Mean		SD	
	Urban	Rural	Urban	Rural
EMO	23.10	24.16	4.85	4.57
COG	25.56	24.29	5.30	5.83
BEH	17.41	17.28	4.01	4.16
SR	66.07	65.73	10.50	10.13

Note. EMO—Emotional dimension; COG—Cognitive dimension; BEH—Behavioral dimension; SR—Self-regulation.

## Data Availability

The results and data can be obtained from the authors.
